# Progress in Otology

**Published:** 1900-05-05

**Authors:** 


### Progress in Otology.

### THE INFLUENCE OF HEREDITY ON DEAFNESS.

Dr. W. Scheppegrell, of New Orleans (U.S.A.) records in the *American Journal of the Medical Sciences* (February, 1900) a series of valuable studies made by him, together with a consideration of a large amount of statistical material, with regard to the above subject. The subject of heredity in relation to deafness and deaf-mutism is one of great importance, and in America it has attracted the serious attention of sociologists and legislators. Bell,[1] who has contributed much to this subject, believes that the evidence which he has collected shows a tendency to support the view of the formation of a deaf variety of the human race in North America. The relation of deafness as an essential and primary factor in deaf-mutism should be borne in mind. "It is not an uncommon case to have a child presented to have its mouth and throat examined, the parents being under the impression that there is some defective development or paralytic condition of these parts, when the real cause is the auditory apparatus," though they must easily be capable of realising that hearing is essential to the normal development of speech, and that the child could not be expected to repeat sounds which it never heard and cannot hear. The degree of deafness, relative or absolute, is of some importance. In some patients the deafness is so complete that no sounds whatever are heard, its proportion among the congenitally deaf having been estimated at about 40 per cent., and among those who have acquired deafness since birth at about 48 per cent. Large and sonorous waves (*e.g.*, deep organ tones) may be perceived by means of the cutaneous nerves; or, according to Egger, in a recent communication before the Société de Biologie[2] by the musculo-periosteal terminations of the deeper (subcutaneous) nerves. When all cases of deaf-mutism in institutions are considered it appears that one-fourth to one-third were congenitally deaf, the remainder having it as an acquired disease. Thus Mygind,[3] from an investigation of 210 cases, found that 110 were acquired and 54 were congenital, the rest being uncertain. The report of the Michigan Asylum for the Deaf and Dumb fixes the proportion of congenital cases at 25 per cent., and of acquired cases at 75 per cent. Objective changes in the auditory apparatus of children reported to be congenitally deaf have been recorded, however, by Politzer[4] and Schmalz,[5] and this serves to cast doubt on the accuracy of the exact ratios of congenital and acquired cases. In the case of children who have acquired deafness at an early age after having learnt such words as "mamma" and "papa" these sounds are subsequently forgotten, and, in fact, "if the deafness develops before the age of seven the speech already acquired gradually becomes less fluent, rougher and less distinct, and unless educational methods are used to overcome the tendency mutism will ultimately result." The commoner causes of acquired deafness are the aural troubles consequent upon scarlet fever, cerebral meningitis, measles, traumatism of the middle ear, syphilis, and other pathological conditions. In different countries the ratio of deaf-mutes to the general population varies. Thus, in Prussia it is 1 in 1,730, in France it is 1 in 1,672, in Great Britain and Ireland it is 1 in 1,636, and in North America it is 1 in 1,251.[6] The lowest average is found in Holland (3·35 per 10,000), and the highest in Switzerland (11·8 per 10,000). The records for various countries (Europe and America) agree that there are more male than female deaf-mutes in the ratio of 11 to 9. Deaf-mutism is relatively common in cretin-producing localities, *e g.*, in several parts of the canton of Berne in Switzerland. A very careful review of returns from various sources show that in the case of deaf mutes marrying, not more than 25 per cent. of their children are born deaf. When only one of the partners is deaf, it was believed that a larger average proportion of deaf children were born. This curious anomaly had, if true, received no satisfactory explanation, and Darwin has stated that, "in the present state of our knowledge it is better to look on the whole case as simply unintelligible." More recent investigations, however, tend to reverse the proportion, and thus to render the problem intelligible. Thus Mygge[7] has shown in a series of investigations that "in cases in which only one parent was a deaf-mute, 6·8 per cent. of the offspring were deaf-mutes; while in cases in which both parents were deaf-mutes the percentage was 13·3, or almost twice as much." In cases where deaf-mutism cannot be traced to heredity, it may be the expression of a condition of degeneracy in the parent, *e.g.*, in association with scrofula and tuberculosis, mental or nervous disease, syphilis, or alcoholism. The special association of deaf-mutism with albinism (Dahl[8]) and with cretinism and goitre (Huth[9]) are of great interest. As a preventive against the increase of deaf-mutism, all marriages between deaf-mutes should be strongly discouraged, or interdicted by law, and this rule should be absolute in the case of deaf-mutes between whom a blood-relationship exists.

[1] Marriages of the Deaf in America (Fay), 1898. [2] Révue Hebdomaire de Laryngologie, Feb. 18, 1899. [3] Politzer's Handbook of Ear Diseases, 1893. [4] Loc. cit. [5] Uber die Taubstummen und ihre Behandlung, Leipzig, 1848. [6] Encyclop. Brittan., 9th ed., art. Deaf and Dumb. [7] Deaf-mutism and Blood-relationship (Danish), Copenhagen, 1879. [8] Albinism and Deaf-mutism (Swedish), 1859. [9] The Marriage of Near Kin, 1875.

